# Addition of proprioceptive neuromuscular facilitation to cardiorespiratory training in patients poststroke: study protocol for a randomized controlled trial

**DOI:** 10.1186/s13063-019-3923-1

**Published:** 2020-02-14

**Authors:** Renata Janaína Pereira de Souza, Daniella Cunha Brandão, José Vicente Martins, Juliana Fernandes, Armele Dornelas de Andrade

**Affiliations:** 1 Departamento de Fisioterapia, Universidade Federal do Rio Grande do Norte (UFRN), Natal, Brazil; 2 Departamento de Fisioterapia, Universidade Federal de Pernambuco (UFPE), Recife, Brazil; 3 Departamento de Fisioterapia, Universidade Federal do Rio de Janeiro (UFRJ), Rio de Janeiro, Brazil

**Keywords:** Clinical trial, Stroke, Breathing exercises, Gait, Respiratory muscles, Rehabilitation

## Abstract

**Background:**

Individuals affected by stroke present with changes in cardiovascular and respiratory functions. Cardiorespiratory training (CRT) is one of the classic intervention guidelines for cardiorespiratory fitness. CRT in association with the proprioceptive neuromuscular facilitation (PNF) technique for respiratory muscles could improve the quality of life, cardiorespiratory function and gait parameters of patients after stroke.

**Objective:**

To assess the effects of respiratory and trunk patterns of CRT associated with PNF on the quality of life, gait, oxygen consumption, respiratory muscle strength and thoracic volumes.

**Methods/design:**

A blind, randomized clinical trial with allocation confidentiality will be performed. Forty patients will be randomized into four groups: CRT-lower limb (LL) plus PNF; CRT-LL and respiration; CRT-upper limb (UL) plus PNF; or CRT-UL and respiration. Individuals will be evaluated at three different times (pretreatment, after 20 days of treatment and 1 month after the end of treatment). The treatment protocol consists of respiratory exercises, 30 min of CRT (cycle ergometer) and then repetition of the respiratory exercises, performed three times a week over a period of 20 days. Primary outcome measures are quality of life, gait, balance, peak oxygen uptake and rib cage compartment volumes. As secondary outcomes, respiratory function and maximal inspiratory and expiratory pressures will be measured.

**Discussion:**

The association of PNF with CRT may be a viable and accessible alternative to increase cardiorespiratory function in patients with stroke.

**Trial registration:**

ClinicalTrials.gov, NCT03171012. Registered on 6 June 2017.

## Background

One of the negative outcomes following the occurrence of a stroke is reduced physical activity [1]. This decrease is a consequence of motor and psychological sequelae after the event [2], which influence the return to activities of daily living [3], work activities and quality of life [1].

Functional deficits are manifested by a lack of control involving several body segments such as the trunk and limb musculature which affect the performance in major and minor movements [4]. In addition, balance and walking speed are diminished in comparison to that expected in healthy individuals of the same age and gender [5], which interferes in activity performance and leads to a functional dependence [6, 7].

The impairment in the skeletal system after stroke affects not only the peripheral muscles but can also involve a decrease in respiratory muscle strength [8], chest wall mobility [9] and postural thorax dysfunction [10]. These impairments together could be related to the decrease in aerobic capacity in this population [11]. Thus, interventions for patients with stroke should be addressed to improve exercise tolerance as well to provide a better adequacy of respiratory muscles.

Respiratory and cardiovascular function are also affected after a stroke [12]. Cardiorespiratory activity is poor in patients with stroke, with peak oxygen uptake (VO_2peak_) measures equivalent to 50–60% of healthy population values for the same age and gender [2]. There is also an increase in the energy cost in daily tasks in those people with functional deficits [5], contributing to reduced social participation [13] and poor quality of life [14]. Maximum oxygen uptake values are also lower than those from healthy individuals [15], as well as other ventilatory parameters such as VO_2peak_ and tidal volume [16].

In addition, this population often has other associated comorbidities such as hypertension and diabetes, which may further delay the recovery of these individuals [17]. Thus, intervention strategies are necessary to interrupt this unfavorable cycle of reduced physical activity, functional capacity decline and increased risk of secondary complications [18].

A home/community physiotherapy program of aerobic and respiratory exercises that could provide increments in the time of active exercise in this population [2] might be attractive to the individual and their family since such a program could reduce the time of intensive rehabilitation and the costs, and restore independence, with a consequent improvement in quality of life [19]. Aerobic exercise training is well known as an effective approach to improve aerobic fitness [20]. Exercises on a cycle ergometer performed for both the upper and lower limbs allow the patient to perform an activity load with a focus on the range of motion, coordination, muscle strengthening and increased exercise tolerance [20, 21]. These exercises are also well tolerated in patients with stroke [22].

As an alternative strategy to stimulate active breathing associated with stretching of the antero-posterior, cranio-caudal and latero-lateral diameters of the trunk, respiratory exercises following the principles of proprioceptive neuromuscular facilitation (PNF) have gained more interest for the treatment of patients with stroke [23, 24]. This approach imposes great awareness of the performed movement on the individual, a factor that is usually not present and which could be helpful in the rehabilitation of patients with stroke [25]. The use of PNF has been traditionally for rehabilitation to address motor impairments related to musculoskeletal parameters such as strengthening and/or stretching of paretic limbs and motor control/motor learning [26]. However, the effectiveness of this technique on respiratory parameters is still little known. In a recent study, respiratory pattern exercises of PNF performed in patients with stroke showed a decrease in bioelectrical activity of the accessory respiratory muscle, which could be related to muscle relaxation and reduction of spasticity and which thus could improve respiratory function [24]. Therefore, we hypothesized that the association of aerobic exercises and exercises with a focus on the respiratory musculature may be a therapeutic alternative capable of improving several functional negative outcomes after a stroke.

The objective of this protocol is to assess the effects of PNF respiratory exercises associated with cardiorespiratory training on respiratory and trunk characteristics, specifically on the quality of life, gait parameters, VO_2peak_, respiratory muscle strength, and thoracic chest wall volumes in patients poststroke.

## Methods/design

This is a prospective, randomized controlled trial with concealed allocation and blinded assessments of patients poststroke (with stroke diagnoses for more than 6 months). Residents living in the community will be recruited from waiting lists of the Physiotherapy Department of the clinical school, as well through disclosures using pamphlets and folders distributed in the community and in social networks including the Communication Agency.

This randomized controlled trial was registered at https://clinicaltrials.gov/ct2/show/NCT03171012 and received approval (65,941,317.7.0000.5537) from the Institutional Ethical Review Board, Universidade Federal do Rio Grande do Norte, Brazil. The study commenced in July 2017 and the estimated completion date is December 2020.  All volunteers will be informed about the objectives, methods and the risks and benefits of the study, and only those who agree to participate by signing an Informed Consent form will be included. The participants will be clearly instructed about the right to refuse to participate in the study at any time of data collection. Intervention procedures and assessment methods do not pose greater risks to volunteers, and will be promptly discontinued in the presence of any reported discomfort. The study protocol was written in accordance with the Standard Protocol Items: Recommendations for Interventional Trials (SPIRIT) guidelines (Additional file 1 shows the SPIRIT checklist). 

### Setting

This study will be carried out in a community-based setting in Recife, Brazil.

### Participants

The inclusion criteria are: primary stroke more than 6 months previously resulting in hemiparesis; age between 21 and 65 years; presence of clinical diagnosis of ischemic or hemorrhagic stroke; absence of cognitive impairment or cognitive deficit (assessed by the Mini-Mental State Examination according to the level of education of participants — less than 19 for illiterate individuals and less than 25 for literate individuals) [27]; being able to walk 10 m independently with or without an assistive device; absence of other neurological or orthopedic deficiencies unrelated to stroke; without reports of associated pulmonary pathology; and not being a current or ex-smoker.

### Participant withdrawal

Patients who miss three consecutive sessions or five intercalated sessions without replacement will be excluded from the study. Also, if the participant reports that they no longer wish to continue they may withdraw from the study at any time.

Participants will be made aware that they are not expected to undergo any other type of physiotherapeutic treatment in order to reduce the risk of interference in the proposed intervention. This requirement will not be enforced in the period between the postintervention evaluation and the follow-up evaluation, but will be recorded if it occurs.

### Randomization procedures

Block randomization was performed through randomization.com, developed by a researcher who is blinded and belongs to the laboratory but who will not participate in other phases of the study. The researcher will place the identification papers of each group in opaque envelopes and seal them, thus maintaining secret allocation. These envelopes will be delivered to the researcher who will perform the interventions only on the first day of therapy.

The participant and the evaluator will be blinded to the type of breathing exercises performed. Furthermore, contact between patients and their interaction and information exchange about respiratory therapy is minimized since they will be attending at scheduled times with intervals between participants. The researcher who will perform the statistical analysis will be blinded to the treatment groups.

### Intervention and control

Twenty sessions will be performed with breathing exercises (spontaneous breathing or using PNF patterns), followed by aerobic exercises for lower or upper limbs, and ending with repetition of the respiratory exercises.

**Fig. 1:**
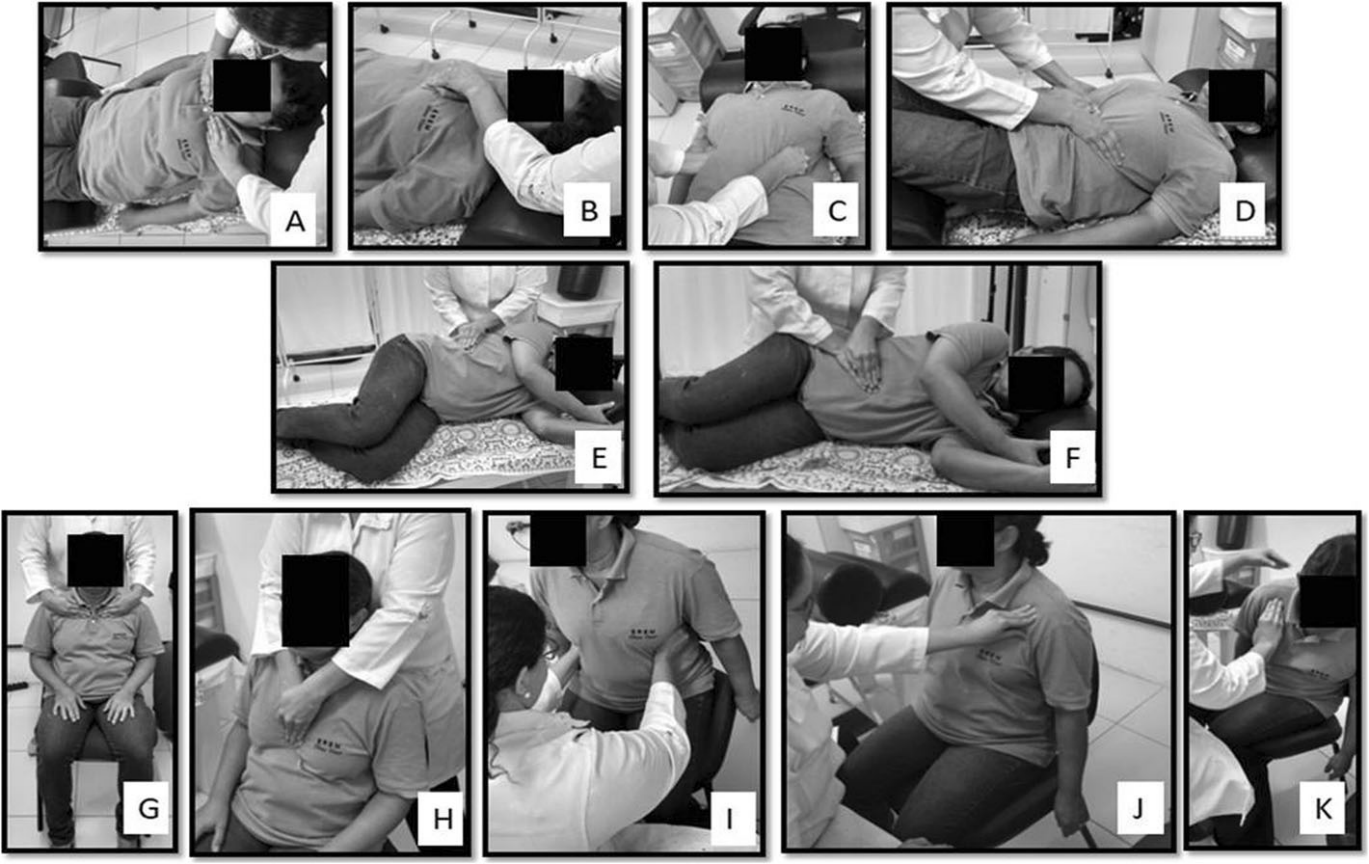
Proprioceptive Neuromuscular Facilitation patterns in experimental group

**Fig. 2:**
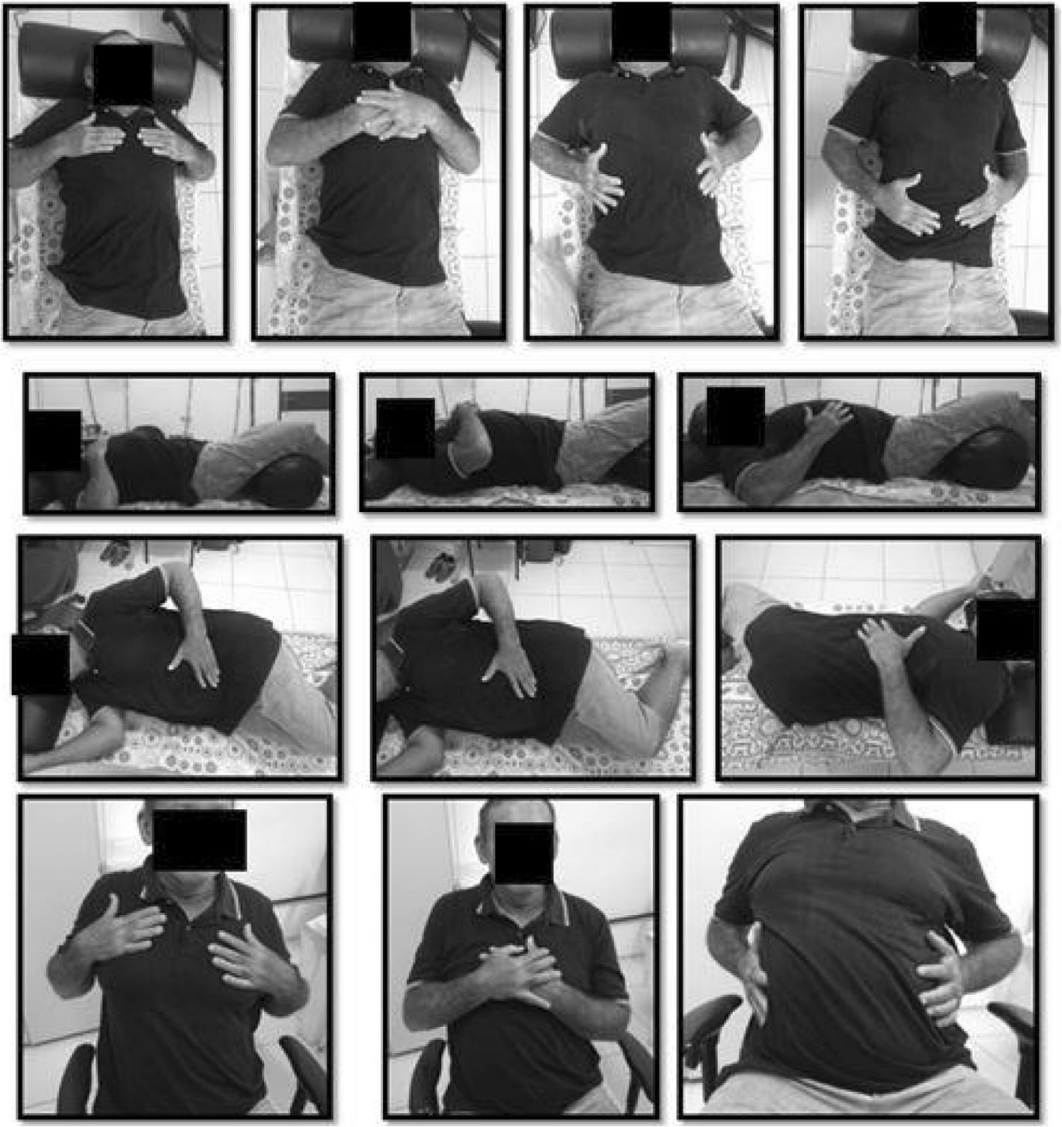
Proprioceptive Neuromuscular Facilitation patterns in control group

The session will begin with the breathing patterns, with each pattern/exercise being performed 10 times. The therapist will position the hands to perform the maneuvers (Fig. 1). In the control group, the patients will put their hands in the same locations used by the therapist during the PNF (Fig. 2).

The cycling will be performed for 30 min per session. Cycling training will be performed without load with a minimum start speed of 50 rpm following the recommendation of the American College of Sports Medicine for patients with stroke [28]. Participants will be encouraged to increase the number of revolutions per minute throughout the study. Thus, participants will perform the cycling at 50 rpm at the beginning; however, there will not be a minimum number of revolutions per minute to reach by the end of the treatment. The patients will be instructed to develop and maintain their highest speed during the exercise, but they may pause if they feel fatigued or need to slow down.

To perform cardiorespiratory training, individuals must be able to remain safe and stable during cycling, with the paretic limb positioned and tied to the pedal. The volunteers will be monitored every 5 min during the 30 min of exercise on the cycle ergometer to check blood pressure, peripheral oxygen saturation and heart rate. The participants will also be asked about their perception of respiratory effort and fatigue in the limbs at the beginning and end of the activity using the original [29] and modified Borg Scale, respectively.

Three evaluations will be carried out at different times: pretraining, post-training and at 30-day follow-up.

The volunteer can be allocated to one of the following protocols:

- Cardiorespiratory training of the lower limbs with respiratory patterns of PNF composed of PNF respiratory exercises patterns, followed by 30 min of exercise on a cycle ergometer carried out by the lower limbs and completed by repeating the PNF exercise breathing patterns.
- Cardiorespiratory training of the upper limbs with respiratory patterns of PNF composed of PNF respiratory exercise patterns, followed by 30 min of exercise on a cycle ergometer carried out by the upper limbs and completed by repeating the PNF exercise breathing patterns.
- Cardiorespiratory training of the lower limbs with spontaneous breathing by the individual composed of spontaneous breathing with the hands positioned at the same points where the hands would be positioned by the therapist during PNF patterns, followed by 30 min of exercises on a cycle ergometer performed by the lower limbs and completed by repeating the initial breathing exercises.
- Cardiorespiratory training of the upper limbs with spontaneous breathing by the individual composed of spontaneous breathing with the hands positioned at the same points where the hands would be positioned by the therapist during PNF patterns, followed by 30 min of exercises on a cycle ergometer performed by the lower limbs and completed by repeating the initial breathing exercises.

During the exercises on the cycle ergometer, the following criteria will be indicated to interrupt the activity:

- Systolic pressure above 140 mmHg [31]
- Diastolic pressure above 100 mmHg
- Peripheral saturation below 90%
- At the request of the volunteer for excessive fatigue or pain

### Experimental group

#### Respiratory patterns of PNF

Respiratory patterns of PNF will be performed in different positions:

1. Supine:
  - Upper costal: both hands of the therapist are positioned laterally on the first four ribs in the hemiclavicular line, resisting the rib movements during inspiration and assisting the ribs to lower during expiration (Fig. 1a).
  - Sternal: both hands of the therapist are positioned on the sternum, resisting the elevation and anterior movements during the inspiration. In the expiratory phase, the therapist assists the movements in the inferior and posterior direction, reducing the antero-posterior diameter of the trunk (Fig. 1b).
  - Therapist's hands: the therapist’s hand is positioned laterally on the trunk, on the four last ribs, resisting the increase of the lateral diameter of the trunk. During expiration the therapist performs movement in the medial and inferior direction (Fig. 1c).
  - Diaphragmatic: the hands of the therapist are positioned on the lower edges of the 8th/9th ribs in both limbs, resisting the lateral and antero-posterior diameter increases during inspiration and performing medial and inferior movements (Fig. 1d).
2. Right/left lateral decubitus:
  - Lower costal: both hands of the therapist are positioned on four superposed last ribs (floating), resisting the increase of the lateral diameter of the trunk. The therapist performs movement in medial and inferior directions during expiration (Fig. 1e).
  - Diaphragmatic: both hands of the therapist are positioned overlapping the lower edges of the 8th/9th ribs, resisting increases in lateral and antero-posterior diameters during inspiration and moving in medial and inferior directions (Fig. 1f).
3. Seated position:

The respiratory patterns in the seated position will follow similar actions proposed for the supine position in the upper costal, sternal and lower costal patterns (Fig. 1g–i).

For the trunk flexion pattern, the hands of the therapist are alternately positioned in the clavicular acromion and scapular spine, creating a support point and resisting trunk flexion and extension movements, respectively. The patient performs a movement of up to 45° of trunk flexion, being instructed to lead the shoulder to the contralateral knee during expiration, rotating the trunk and overcoming the resistance imposed by the therapist. In extension, the individual takes the inspiration and returns to the position of 90°, with resistance in the super-posterior region being guided by the therapist (Fig. 1j and k).

The technique used was stabilization reversal. The patterns are performed in both the most affected and contralateral regions. During PNF application, patients are instructed to breathe in deeply, and they are in control of the onset of inspiratory movement. The therapist will count the respiratory pattern of the patient, and they will be instructed to stop the exercise after 10 respirations have been performed.

#### Measurements

The evaluations will be carried out by a trained researcher who is blinded to the treatment received by the participant. The evaluations will be divided over 2 days to avoid fatigue of the participant. The assessment process will be composed of the Mini-Mental State Examination, anamnesis, plethysmography optoelectronics, manovacuometry, spirometry, a 6-min walk test (6MWT; with inertial walking sensor and gas analyzer), quality of life scale, and Berg balance scale. The database will be populated by the researcher responsible for the evaluation, with the process being performed twice to minimize errors.

#### Primary outcome measures

The primary outcomes are oxygen uptake (VO_2_), balance, quality of life, gait parameters (walking distance, step length, stance and swing time) and rib cage compartment volumes.

Oxygen uptake will be measured during a 6MWT. This test will be performed in a 30-m hallway by a trained therapist following the guidelines proposed by the American Thoracic Society [32]. The 6MWT is validated for patients with stroke [33]; in addition, the VO_2_ obtained in this test presented good validity and reliability with maximum VO_2_ [34]. During the 6MWT participants will be using a telemetry system (MetaMax® 3B, Cortex Biophysik GmbH, Germany) where exhaled gas kinetics (VO_2_, carbon dioxide production (VCO_2_) and the respiratory exchange ratio) will be measured breath-by-breath. The total distance covered and the perception of self-reported effort (Borg Scale, VO_2_, VCO_2_, minute ventilation (VE) and heart rate) will be recorded [32, 35]. The average of the last 30 s of data from the test will be used to determine VO_2peak_, VCO_2_, the respiratory exchange ratio and VE [36].

Balance will be evaluated through the Brazilian version of the Berg Balance Scale (BBS) [37]. BBS evaluates balance based on 14 common activities of daily living items. Each item of the scale presents an alternative of an ordinal scale, varying from 0 to 4 points. The maximum score that can be reached is 56, with a cut-off point of 45 for a risk of falls.

The quality of life evaluation will be evaluated through the stroke-specific quality-of-life scale developed by Williams et al. in 1999 [38] and adapted to the Brazilian population [39]. This is considered a valid measurement scale with adequate reliability (intraclass correlation coefficient, 0.97) for specific evaluation of the quality of life after stroke. It is composed of 49 items distributed in 12 domains. Responses are quantified on a scale that can range from 1 to 5 points and refer to the individual’s performance in the previous 2 weeks. The total score varies from 49 to 245 points, being determined by a greater or lesser dependence and difficulty in performing tasks. The evaluation will be conducted through an interview to avoid a low education level of some participants causing difficulties in answering the questions.

For gait parameters, in addition to collecting expired gases during the 6MWT, the participants will also use the G-walk inertial sensor (BTS Bioengineering, Milan, Italy) to collect the following parameters: walking distance, step length, stance and swing time. The BTS G-SENSOR 2 is a wireless system with an inertial sensor consisting of a three-axis accelerometer, a magnetic sensor and a three-axis gyroscope, which is positioned in L5 for a functional gait analysis. The system calculates all the temporal and spatial gait parameters required to perform a diagnosis or to define a training strategy from the obtained data.

For measurement of the compartmental distribution of the trunk volume volunteers will be referred after an initial evaluation for an analysis of the chest wall kinematics through an optical-electronic plethysmograph (BTS Bioengineering, Milan, Italy) with eight cameras (four anterior and four posterior). According to the protocol of Aliverti and Pedotti [40], 89 reflexive markers will be fixed with hypoallergenic bioadhesive on the anterolateral and posterior faces of the trunk in a grid system. Volunteers will be seated with their feet flat on the floor, knees and hips at 90°, with an erect spine with hands resting on their hips, and then asked to perform quiet breathing without speaking and without changing their posture during the image acquisition.

#### Secondary outcome measures

Respiratory function will be assessed using a Koko spirometer with which the following variables will be measured: inspiratory capacity, forced expiratory volume in 1 s (FEV1), forced vital capacity (FVC), FEV1/FVC, and peak expiratory flow. Spirometry will be performed with the participants in a seated position, with feet resting on the floor, an erect spine, without restraints on the upper limbs and using a mouthpiece and nasal clip. At least three FVC and slow vital capacity maneuvers will be performed, with a 2-min interval between maneuvers according to the American Thoracic Society reproducibility and acceptability criteria [41] and the guidelines for function testing [42], in which a variation of 0.2 l between the tests and the average of the three measurements will be considered [43]. The spirometric values will be expressed as the percentage of the predicted normal value for the Brazilian population [44].

An MV-330 model manovacuometer (Marshal Town Instrumentation Industries, USA) will be used to measure maximal inspiratory and expiratory pressures. The participants will be in a seated position, with feet supported on the floor, an upright spine, without support for the upper limbs and using a mouthpiece and nasal clip, and will be instructed to perform a maximum and sustained inspiration to measure the maximal inspiratory pressure from the residual volume, and perform a maximum expiration to measure the maximal expiratory pressure from the total lung capacity [45]. The best among three maneuvers will be used to record the data.

The oxygen uptake efficiency slope (OUES) represents a relationship between VO_2_ and VE. It is a logarithm of the ventilatory equivalent of oxygen. The OUES can be obtained using the equation (VO_2_ = *a* log VE + *b*). In this equation, the constant *a* represents the regression coefficient (called the OUES), and *b* represents the intercept. OUES correlates with maximum VO_2_ and can predict respiratory capacity during exercise as well as exercise tolerance in patients with various pathologies without the need for maximum effort [46, 47].

#### Other exploratory outcomes and covariates

For anthropometric and body composition evaluation, the weight and height will be measured by means of a digital scale with a stadiometer (Welmy model W300; São Paulo, Brazil) with a capacity of 300 kg, an accuracy of 50 g and a stadiometer with limit of 2 m. Body mass index will be calculated by dividing the body weight by the height squared (kg/m^2^). Trunk muscle mass and the upper and lower limbs will be evaluated using the InBody R20 electrical impedance scale (Biospace Co. Ltd., Korea), which uses a low-amplitude, high-frequency electric current to calculate the percentages and totals of water and fat in the body, and the metabolic rate.

Cardiopulmonary evaluation will be measured by the peripheral oxygen saturation, heart rate, systolic and diastolic blood pressures and the respiratory rate using a DIXTAL 2023 multiparameter monitor (Manaus, Brazil).

### Sample size calculation

The sample size was calculated using the G*Power Program (Version 3.1.9.2, University of Kiel, Germany) and the variable VO_2peak_ (in liters per minute) was adopted as the primary outcome measure. According to a previous study involving individuals with poststroke hemiparesis who performed cycling training [48], standard deviations observed in the experimental and control groups were 0.16 and 0.13, respectively. The sample size was calculated from these data to detect a difference in VO_2peak_ between the groups after intervention of 0.24 l/min (α = 5% and power = 80%), using a two-sample *t* test for mean difference. The sample required was eight patients; considering a follow-up loss of 20%, 10 individuals will be allocated into each intervention group: 10 individuals in the experimental lower limb group (cardiorespiratory training of the lower limbs plus PNF), 10 individuals in the control lower limb group (cardiorespiratory training of the lower limbs plus breathing), 10 individuals in the experimental upper limb group (cardiorespiratory training of the upper limbs plus PNF) and 10 individuals in the control upper limb group (cardiorespiratory training of the upper limbs plus breathing).

### Statistical analyses

A descriptive analysis of the data will be initially performed. Frequency distribution will be obtained for the categorical variables, and central tendency and dispersion measures will be obtained for the numerical variables. The Kolmogorov-Smirnov/Shapiro-Wilk test and the Levene test will be used to evaluate the normality of the variables and the homogeneity of the sample, respectively.

All tests will be applied assuming a 95% confidence level, considering a significant *P* value <0.05. An intention-to-treat analysis will be performed in case of patient withdrawal or interruption of training, using the last observation carried forward. Two-way repeated-measures analysis of variance to determine the interaction intervention by time (pretraining, post-training and 30 days of follow up) for the outcome variables will be conducted for the experimental and control lower limb interventions and the experimental and control upper limb interventions separately. Paired *t* tests will be used to compare clinical outcomes between preintervention and post-training for experimental and control groups. Independent *t* tests will be also used to separately compare upper limb experimental and control groups and lower limb experimental and control groups post-training.

## Discussion

### Relevance of the study

The purpose of this clinical trial is to evaluate the efficacy of the addition of PNF patterns performed on the trunk to cardiorespiratory training in patients with stroke. Although there are a number of studies, including systematic reviews [1, 20], which clarify the need to increase the frequency of physical exercise in this population or discuss exclusive training for respiratory muscles [49], the union of these therapies is still not well documented in the literature.

The focus of treatment on respiratory muscles through PNF respiratory patterns provides an innovative approach for patients with stroke undergoing cardiorespiratory rehabilitation. In our protocol, stabilization reversal which is characterized by alternating isotonic contractions opposed by resistance to prevent motion, will be used as a specific PNF technique. We will apply these techniques in several positions, both seated and supine. The patterns of PNF associated with respiration will be performed 10 times for both PNF and control groups.

To date, we have found only one study that applied PNF respiratory patterns in patients with stroke; however, this study focused on electromyography activities, and information about the influence of PNF techniques on aerobic capacity, pulmonary function and chest wall distribution is still lacking.

Thus, we believe that our protocol will improve the current literature since the association of PNF therapy and cycle ergometer training could provide a more affordable therapy, and is feasible for use in the early stages and in various therapeutic settings to improve respiratory function.

### Trial status

This is protocol version 2. This trail was prospectively registered at ClinicalTrials.gov (NCT03171012) on 6 June 2017. Recruitment began in June 2017 and is ongoing. The anticipated completion date is December 2020.

## Supplementary information

**Additional file 1.** SPIRIT check list.

## Abbreviations

6MWT: Six-minute walk test
BBS: Berg Balance Scale
FEV1: Forced expiratory volume in 1 s
FVC: Forced vital capacity
OUES: Oxygen uptake efficiency slope
PNF: Proprioceptive neuromuscular facilitation
VCO_2_: Carbon dioxide production
VE: Minute ventilation
VO_2_: Oxygen uptake
VO_2peak_: Peak oxygen uptake
